# Dynamic relationships between microbial biomass, respiration, inorganic nutrients and enzyme activities: informing enzyme-based decomposition models

**DOI:** 10.3389/fmicb.2013.00223

**Published:** 2013-08-12

**Authors:** D. L. Moorhead, Z. L. Rinkes, R. L. Sinsabaugh, M. N. Weintraub

**Affiliations:** ^1^Department of Environmental Sciences, University of ToledoToledo, OH, USA; ^2^Department of Biology, University of New MexicoAlbuquerque, NM, USA

**Keywords:** extracellular enzymes, models, decomposition, soil microorganisms, enzyme efficiency

## Abstract

We re-examined data from a recent litter decay study to determine if additional insights could be gained to inform decomposition modeling. Rinkes et al. (2013) conducted 14-day laboratory incubations of sugar maple (*Acer saccharum*) or white oak (*Quercus alba*) leaves, mixed with sand (0.4% organic C content) or loam (4.1% organic C). They measured microbial biomass C, carbon dioxide efflux, soil ammonium, nitrate, and phosphate concentrations, and β-glucosidase (BG), β-N-acetyl-glucosaminidase (NAG), and acid phosphatase (AP) activities on days 1, 3, and 14. Analyses of relationships among variables yielded different insights than original analyses of individual variables. For example, although respiration rates per g soil were higher for loam than sand, rates per g soil C were actually higher for sand than loam, and rates per g microbial C showed little difference between treatments. Microbial biomass C peaked on day 3 when biomass-specific activities of enzymes were lowest, suggesting uptake of litter C without extracellular hydrolysis. This result refuted a common model assumption that all enzyme production is constitutive and thus proportional to biomass, and/or indicated that part of litter decay is independent of enzyme activity. The length and angle of vectors defined by ratios of enzyme activities (BG/NAG vs. BG/AP) represent relative microbial investments in C (length), and N and P (angle) acquiring enzymes. Shorter lengths on day 3 suggested low C limitation, whereas greater lengths on day 14 suggested an increase in C limitation with decay. The soils and litter in this study generally had stronger P limitation (angles >45°). Reductions in vector angles to <45° for sand by day 14 suggested a shift to N limitation. These relational variables inform enzyme-based models, and are usually much less ambiguous when obtained from a single study in which measurements were made on the same samples than when extrapolated from separate studies.

## Introduction

Decomposition occupies a central position in global biogeochemical cycles and mathematical models play a central role in efforts to understand them and predict future changes. Decomposition models span a wide range of temporal, spatial, and hierarchical scales of resolution (Manzoni and Porporato, 2009), from physiologically based simulations of microbial activity in laboratory cultures (Resat et al., 2012) to empirical models that estimate gas flux dynamics over regional landscapes (Niu et al., 2012). The scale of interest necessarily defines the resolution of the appropriate model (Reynolds and Leadley, 1992). In any case, decomposition of the most common structural polymers comprising dead organic matter, i.e., cellulose, hemicellulose, and lignin, is largely accomplished at the biochemical level by the activities of extracellular enzymes produced by microorganisms (Burns, 1983; Sinsabaugh, 1994). Thus representative models minimally require detailed information about interactions between microorganisms, their extracellular enzymes, and substrates they degrade. Fortunately, studies of enzyme activity in the environment have expanded rapidly over the last few years [see review by Burns et al. (2013)] and enzyme-based models are beginning to emerge (Schimel and Weintraub, 2003; Allison, 2005; Moorhead et al., 2012; Resat et al., 2012; Wang et al., 2013). Unfortunately, the data needed to develop and test these models are incomplete and often gleaned piecemeal from disparate studies which raises questions about cross study comparisons. Herein we briefly review the development of enzyme-based decomposition models, highlight common information gaps, and demonstrate the contribution to modeling objectives obtained from closely integrated studies of the substrate-enzyme-microbe (SEM) system during decomposition.

## Background

### Enzyme models

The first models to link enzyme activity to decomposition were the statistically based enzyme decay models (EDMs) that regressed litter mass loss against cumulative measures of enzyme activities (Sinsabaugh, 1994; Jackson et al., 1995). These models demonstrated that the activities of enzymes that hydrolyze related groups of compounds, like cellulose and hemicellulose, correlate with each other. Thus a single indicator enzyme could be used as a proxy for the combined activities of a suite of enzymes that degrade a particular substrate, such as β-glucosidase (BG) for holocellulose, β-N-acetyl-glucosaminidase (NAG) for chitin and peptidoglycan, leucine amino-peptidase (LAP) for proteins, and acid or alkaline phosphatase (AP) for organic P. More recently, a synthesis of collected measurements from soils, sediments, and freshwater plankton (Sinsabaugh and Follstad Shah, 2012) showed that the patterns of activities for key indicator enzymes (i.e., BG, NAG, LAP, and AP) integrated the metabolic and stoichiometric requirements of decomposer organisms with the relative availabilities of C, N, and P from environmental sources. This enzymatic stoichiometry theory (EST) provides a rationale for mechanistic models linking enzyme activity to decomposition, but is based on observations usually including key enzyme activities and total N, P, and organic C pool sizes, and seldom includes microbial biomass or metabolic activity, such as respiration. For this reason, EST describes the overall phenomenon but not the mechanisms of SEM interactions (Reynolds and Leadley, 1992).

**Figure 1:**
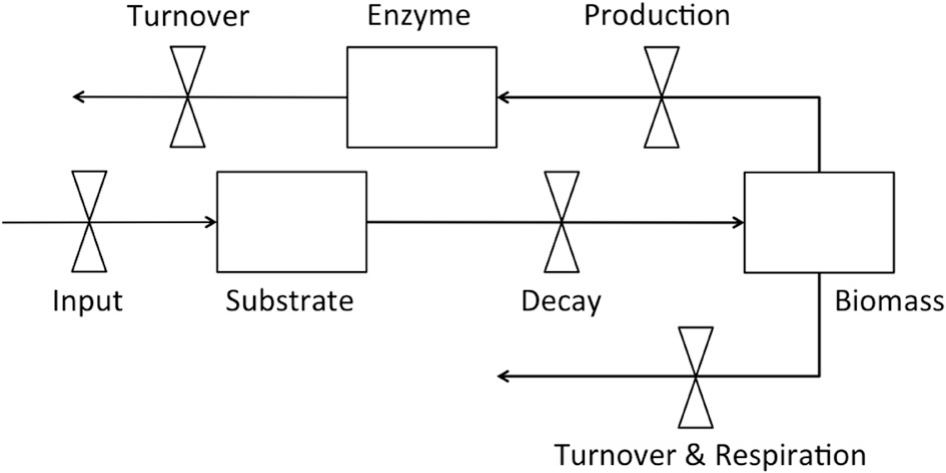
**Flow diagram of a simple enzyme-based decomposition model**.

Dynamic enzyme-based models typically include explicit pools of enzymes, microbial biomass, and substrate (Figure 1) with substrate decomposition providing resources supporting biomass and enzyme production, as well as respiration (Sinsabaugh and Moorhead, 1997; Vetter et al., 1998; Schimel and Weintraub, 2003; Allison, 2005, 2012; Allison et al., 2010, 2011; Folse and Allison, 2012; Moorhead et al., 2012; Resat et al., 2012; Wang et al., 2013). Turnover of microbial biomass and enzymes is commonly needed to balance these pools with substrate input. However, data on turnover rates are rarely published (but see Allison, 2006). Even the simple model in Figure 1 requires information that is seldom available, and incorporates hypothetical relationships that are controversial. For example few experimental studies have simultaneously examined the dynamics of microbial biomass, enzymes, and substrate, so instead, feedback controls necessary to balance the relative SEM relationships, such as production and turnover rates, are often “fit” to maintain model stability (e.g., Sinsabaugh and Moorhead, 1997; Schimel and Weintraub, 2003; Lawrence et al., 2009). Even the relative flows of resources to enzymes, biomass, and respiration are uncertain, depending on which model of carbon use efficiency is employed (Wang and Post, 2012; Sinsabaugh et al., 2013; Wang et al., 2013), and whether enzyme production is constitutive, inducible, or both (Schimel and Weintraub, 2003; Allison, 2005). Finally, the enzyme pool size is never measured directly, and instead potential activity is assumed to be proportional to the concentration of enzyme.

More complex models simulate the degradation of different substrates by different types of enzymes, but retain all the limitations of simpler models while adding more pools and associated uncertainties about multiple SEM interactions (Allison, 2005; Folse and Allison, 2012; Moorhead et al., 2012). Sinsabaugh and Follstad Shah (2012) argued that patterns of enzyme activity reflect microbial metabolic and stoichiometric needs, limited by patterns of resource availability. This general rationale integrated energy and nutrient controls in decomposition models long before enzymes were explicitly included (e.g., Parnas, 1975; Parton et al., 1987; Skjemstad et al., 2004). More recently, Moorhead et al. (2012) used this rationale to derive an analytical solution for the optimum allocation of C and N acquiring enzyme activities by decomposer microorganisms. Similarly, Allison (2005) assumed that enzyme production was induced by resource deficits and allocated this production among C, N, and P acquiring enzymes to balance microbial requirements. Both of the latter models also retained the uncertainties of simpler enzyme-based models despite generating relative patterns of different enzyme activities that could be compared to experimental studies. Thus, many of the underlying assumptions built into enzyme-based models remain untested.

### Information gaps

In general, enzyme-based models simulate the flow of carbon and nutrients between pools that are often difficult to empirically quantify or test. Nonetheless, any effort to incorporate first-principle mechanisms driving soil organic matter dynamics must address the fundamental relationships within the SEM system, including the basic physiology of decomposer microorganisms and the dynamics of their extracellular enzymes. Most experimental studies to date have limited power to support or test key assumptions of these models. For example, microbial biomass is rarely monitored during decomposition, and the dynamic interaction between biomass and system carbon (Figure 1) is unclear. Microbial biomass is usually reported to be low, seldom exceeding 2–3% of the total organic C pool in soils (Anderson and Domsch, 1989; Wardle, 1998), but the mechanisms controlling biomass are poorly defined. Without measures of microbial biomass, we cannot directly relate enzyme activities and CO_2_ efflux to the microbial pool responsible for their production.

Even when microbial biomass is monitored closely, other aspects of decomposition are often omitted. For example, Kuehn et al. (2000), Gessner (2001), and Suberkropp (2001) all monitored microbial biomass dynamics on decomposing litter in aquatic ecosystems. However, none of these studies measured extracellular enzyme activities. In contrast, a suite of detailed studies examining litter decay in a Mediterranean ecosystem closely monitored microbial biomass, enzyme activities, litter mass and chemistry, and respiration (Fioretto et al., 2000, 2001, 2003, 2005, 2007). However, their focus was primarily on C dynamics and they measured neither N nor P acquiring enzyme activities. Also, Fioretto et al. (2007) found that high seasonal variation in moisture stress produced high seasonal variation in enzyme activities that decoupled apparent activities from litter mass loss. Thus physical variations can obscure biological relationships. In short, few studies have obtained the basic measurements needed to develop or test enzyme-based decomposition models.

Here we examine observations made during a detailed study of litter decomposition in laboratory microcosms, including simultaneous measures of enzyme activities, microbial biomass, and CO_2_ efflux. Our goals are to (1) analyze these data from the perspective of model requirements, i.e., for relationships between variables in comparison to separate analyses of independent variables, and thus (2) illustrate how simultaneous measures of these metrics during decomposition can provide insights to relationships needed to inform enzyme-based models.

## Case study with alternative analyses

We use new analyses of data reported by Rinkes et al. (2013) as a case study of how relationships among simultaneous measures of enzyme activities, microbial biomass, and CO_2_ efflux can be useful for decomposition modeling (Figure 1). In brief, they conducted a short-term (14 days) laboratory study of litter decomposition to evaluate the relationships between CO_2_ efflux, microbial C, extracellular enzyme activities, and changes in soil mineral N and P concentrations during the initial onset of litter decay. Their explicit goals were to examine (1) the effects of soil type, and both litter quality and surface area; and (2) the interactions between litter decay and the organic matter “priming effect” at the start of decomposition. To these ends, 1 g of sugar maple (*Acer saccharum*) or white oak (*Quercus alba*) leaves were cut to one of three sizes (ground, 0.25 cm^2^, and 1.0 cm^2^), and mixed with 50 g of either a sandy soil with low soil organic C (SOC) content (0.4%) or a loam with moderate SOC content (4.1%), wetted to 45% water-holding capacity (WHC) and incubated at 20°C. Soils were first pre-incubated for 5 months in a dark 20°C incubator at 45% WHC. The pre-incubation allowed for microorganisms to acclimate to experimental conditions and to metabolize as much extant labile C as possible, in order to better isolate the specific response of litter additions. Microbial biomass C, soil NH^+^_4_, NO^−^_3_, and PO^−3^_4_ concentrations, and β-glucosidase (BG), β-N-acetyl-glucosaminidase (NAG), and acid phosphatase (AP) activities were measured on days 0 (initial values), 3 and 14; CO_2_ efflux was measured on days 1, 2, 3, 4, 6, 7, and 14.

Rinkes et al. (2013) discussed the primary results of their experiment in detail, but models require specific types of related information not reported in their study, such as CO_2_ efflux and enzyme activity per unit microbial biomass. Thus we analyzed their data differently to achieve a separate set of goals. Rinkes et al. (2013) were interested in the effects of litter surface area on decomposition and had 4 replicates of each litter size class (3 sizes) for each combination of litter (2 l) and soil type (2 soils). This provided three means (*N* = 4) for each measured system parameter for each combination of soil and litter type by date. However, Rinkes et al. (2013) did not link the CO_2_ efflux measurements to the same microcosm replicates as other system measurements. As a result, CO_2_ efflux could not be paired to enzyme activity or soil nutrient content by replicate. Instead, mean C fluxes from a particular combination of litter type, litter size and soil type for a date were compared to the means of the other system measures that is there were three means per date for each system parameter, defined by litter particle size class, and litter and soil type. We limited our attention to CO_2_ efflux rates on days 1, 3, and 14, which corresponded to the timing of measures taken for other system parameters. Finally, we did not subtract values of CO_2_ efflux and enzyme activities observed in soil-only controls from litter addition treatments as Rinkes et al. (2013) did, because we were interested in total system behaviors and subtracting control values from treatment values emphasizes litter dynamics, alone.

Our primary interest in using these data was to explore direct relationships between microbial dynamics, enzyme activities, and litter decay, and how they varied with soil and litter characteristics. Thus we calculated total organic carbon (TOC = SOC + litter C) through time. All incubations began with 1 g litter (44% C for both oak and maple) and 50 g soil, but sand had 0.4% SOC content whereas loam had 4.1% SOC, resulting in 0.64 and 2.49 g TOC, respectively. CO_2_ efflux rates were subtracted from TOC over time to estimate remaining C per unique replicate (*N* = 4 by day, litter size, soil, and litter type). We then calculated three mean values (one for each litter particle size) of CO_2_ efflux and TOC for each combination of day, litter, and soil type that could then be compared to the mean values of other system characteristics, e.g., enzyme activities, biomass, and soil nutrient concentrations. We also assumed that CO_2_ efflux measures on day 1 corresponded to initial biomass, nutrient, and enzyme measures on day 0, although they were taken over 24 h from the start of the experiment, and thus likely overestimate CO_2_ efflux rate per unit biomass because the biomass was growing (reported below).

The statistical analyses performed by Rinkes et al. (2013) usually examined the effects of litter particle size, litter type, and soil type on system characteristics, e.g., CO_2_ efflux, enzyme activity, and mineral nutrients. However, our focus was not on litter particle size, so we analyzed the three litter particle size classes together, which reduced most of our analyses to Two-Way ANOVAs (litter and soil), with separate analyses conducted for each day. These differences in statistical design often resulted in differences between studies. In particular, significant effects of litter particle size, or interactions between particle size and other main effects were not apparent in our analyses. This often led to different conclusions about the contributions of the other independent factors (below) and illustrates the potential impact empirical studies could have on decomposition models by including additional analyses that explicitly address modeling needs.

### Carbon flux rates

C flux rates can be expressed in several ways. For example, Rinkes et al. (2013) reported CO_2_ efflux per g soil but subtracted soil-only control values from treatments. We did not subtract control values and also estimated rates per g TOC and per g microbial biomass because activities per unit microbial biomass are necessary to develop and test relationships in mechanistic models (Figure 1). For this reason we examined microbial biomass (μg C·g soil^−1^) by day, soil, and litter type, and found that it was initially greater (day 0) for loam than sand (Table 1). However, by day 3 there were no differences between treatments, but on day 14, biomass was again higher for loam than sand. Rinkes et al. (2013) noted that microbial biomass was initially greater for loam than sand controls, but there were no differences between treatments at any time after soil-only control values were subtracted. They also reported that total (uncorrected) biomass increased from day 0 to day 3 (values between 140–300 μg C·g soil^−1^) and generally decreased from day 3 to day 14. Thus our results are consistent with the few observations they reported.

**Table 1:** **Results of Two-Way ANOVA of independent and relational variables for soil and litter types were based on data collected by Rinkes et al. (2013)**.

**Factor**	**Units**	**Day**	**Loam**	**Sand**	**Maple**	**Oak**
Microbiota	μg C·g soil^−1^	0	143.7 ± 98.4^**^	22.7 ± 27.9^**^	107.0 ± 116.2	59.5 ± 63.5
		3	356.1 ± 175.4	315.1 ± 248.9	334.2 ± 224.6	337.0 ± 207.7
		14	208.6 ± 75.7^**^	114.8 ± 107.6^**^	158.3 ± 102.9	164.8 ± 106.1
Microbiota	mg C·g C^−1^	0	2.94 ± 0.81	1.81 ± 2.22	3.45 ± 0.10	1.31 ± 1.50
		3	7.33 ± 2.34^**^	25.49 ± 2.73^**^	16.51 ± 10.57	16.31 ± 9.96
		14	4.38 ± 0.69	9.51 ± 6.85	6.64 ± 4.65	7.25 ± 6.42
CO_2_ efflux	μg C·g soil^−1^·d^−1^	1	100.70 ± 68.95^**^	25.61 ± 10.06^**^	70.10 ± 67.42	56.21 ± 56.16
		3	232.70 ± 46.46^**^	167.16 ± 33.17^**^	229.78 ± 45.82^**^	172.70 ± 41.62^**^
		14	67.73 ± 6.67^**^	39.77 ± 9.96^**^	56.95 ± 16.68	51.82 ± 16.06
CO_2_ efflux	mg C·g C^−1^·d^−1^	1	2.07 ± 1.41	2.03 ± 0.77	2.26 ± 1.14	1.84 ± 1.09
		3	4.80 ± 0.96^**^	13.54 ± 2.75^**^	10.35 ± 5.40^**^	7.86 ± 4.01^**^
		14	1.41 ± 0.15^**^	3.32 ± 0.79^**^	2.34 ± 1.00	2.30 ± 1.24
CO_2_ efflux	g C·g Biomass C^−1^·d^−1^	1	0.713 ± 0.521	4.033 ± 4.370	0.661 ± 0.328^*^	4.085 ± 4.340^*^
		3	0.713 ± 0.291	0.534 ± 0.102	0.725 ± 0.263	0.522 ± 0.142
		14	0.328 ± 0.049	0.494 ± 0.280	0.439 ± 0.255	0.383 ± 0.173
∑CO_2_ efflux	mg C·g soil^−1^	1	5.14 ± 3.51^*^	1.30 ± 0.49^*^	3.57 ± 3.45	2.88 ± 2.86
		3	22.98 ± 7.10^**^	17.21 ± 5.60^**^	23.19 ± 5.95^**^	17.25 ± 6.74^**^
		14	73.03 ± 11.00^**^	45.95 ± 6.85^**^	66.81 ± 15.17^**^	53.40 ± 15.19^**^
Total C	mg C	0	2.490^a^	0.640	2.490	0.640
		3	2.477 ± 0.006^**^	0.630 ± 0.005^**^	1.552 ± 0.944^*^	1.555 ± 0.943^*^
		14	2.441 ± 0.010^**^	0.608 ± 0.007^**^	1.603 ± 0.934^**^	1.530 ± 0.939^**^
Litter C	g C	0	0.440	0.440	0.440	0.440
		3	0.427 ± 0.006§	0.430 ± 0.005§	0.427 ± 0.006^*^	0.430 ± 0.005^*^
		14	0.391 ± 0.010^**^	0.408 ± 0.007^**^	0.394 ± 0.010^**^	0.405 ± 0.011^**^
AP activity	nmol·g soil^−1^·h^−1^	0	613.6 ± 81.9^**^	152.0 ± 68.3^**^	349.6 ± 223.3§	416.1 ± 284.2§
		3	841.0 ± 201.1^**^	158.1 ± 99.5^**^	418.6 ± 332.9^**^	580.5 ± 411.2^**^
		14	737.9 ± 262.2^**^	100.3 ± 23.3^**^	384.6 ± 304.9§	465.3 ± 429.6§
NAG activity	nmol·g soil^−1^·h^−1^	0	276.8 ± 29.8^**^	40.6 ± 10.3^**^	152.9 ± 115.8	164.6 ± 139.2
		3	337.0 ± 56.1^**^	121.5 ± 80.6^**^	254.2 ± 112.3^*^	204.3 ± 141.3^*^
		14	543.8 ± 293.4^**^	320.3 ± 128.0^**^	510.4 ± 238.2^*^	361.6 ± 248.4^*^
BG activity	nmol·g soil^−1^·h^−1^	0	165.8 ± 35.9^**^	80.9 ± 31.4^**^	145.0 ± 54.5^**^	101.7 ± 48.6^**^
		3	86.8 ± 23.6^**^	33.2 ± 20.3^**^	72.7 ± 34.1^**^	47.3 ± 31.2^**^
		14	818.1 ± 436.6^**^	496.4 ± 142.1^**^	744.3 ± 352.8	580.5 ± 360.5
AP activity	μmol·g Biomass^−1^·h^−1^	0	693.19 ± 1947.32	682.63 ± 1035.33	694.91 ± 1946.65	680.91 ± 1036.57
		3	2.94 ± 1.44^**^	0.92 ± 0.93^**^	1.68 ± 1.60	2.19 ± 1.54
		14	4.37 ± 3.57	2.88 ± 4.40	2.74 ± 2.39	4.50 ± 5.04
NAG activity	μmol·g Biomass^−1^·h^−1^	0	339.52 ± 954.51	143.43 ± 199.26	339.42 ± 954.55	143.54 ± 199.16
		3	1.23 ± 0.75	0.83 ± 1.00	1.24 ± 1.04§	0.82 ± 0.69§
		14	3.13 ± 2.18§	9.23 ± 15.09§	6.04 ± 8.09	6.19 ± 13.37
BG activity	μmol·g Biomass^−1^·h^−1^	0	258.26 ± 727.29	238.78 ± 349.57	260.21 ± 726.52	236.83 ± 351.05
		3	0.33 ± 0.28	0.24 ± 0.31	0.38 ± 0.37^*^	0.19 ± 0.15^*^
		14	4.85 ± 3.46§	13.84 ± 20.43§	9.92 ± 16.61	8.61 ± 13.69
PO^−3^_4_	μg P·g soil^−1^	0	7.93 ± 7.85^**^	37.77 ± 18.53^**^	29.07 ± 20.71§	16.62 ± 19.90§
		3	0.35 ± 0.45^**^	5.23 ± 4.93^**^	4.53 ± 4.53^**^	1.05 ± 3.17^**^
		14	0.11 ± 0.07^**^	2.44 ± 2.35^**^	2.07 ± 2.28^**^	0.48 ± 1.36^**^
NO^3−^	μg N·g soil^−1^	0	163.55 ± 15.13^**^	13.76 ± 0.96^**^	88.40 ± 81.16	88.91 ± 80.40
		3	144.67 ± 19.06^**^	4.03 ± 2.92^**^	73.56 ± 71.85	75.14 ± 74.35
		14	94.92 ± 28.36^**^	0.97 ± 1.01^**^	47.19 ± 50.14	48.70 ± 53.81
NH^4+^	μg N·g soil^−1^	0	24.72 ± 8.03^**^	2.60 ± 0.85^**^	10.87 ± 8.58^**^	16.46 ± 15.92^**^
		3	21.83 ± 10.28^**^	0.10(trace)^**^	8.31 ± 11.62^**^	13.62 ± 14.23^**^
		14	1.81 ± 0.29^**^	0.26 ± 0.15^**^	0.96 ± 0.84^*^	1.11 ± 0.80^*^
Vector length	Unitless	0	0.665 ± 0.155^**^	2.058 ± 0.515^**^	1.596 ± 0.896^**^	1.126 ± 0.685^**^
		3	0.280 ± 0.069^**^	0.459 ± 0.250^**^	0.464 ± 0.246^**^	0.275 ± 0.067^**^
		14	2.886 ± 4.931^**^	5.604 ± 2.004^**^	4.358 ± 2.548^**^	4.080 ± 5.067^**^
Vector angle	Degrees	0	65.54 ± 3.09^**^	73.40 ± 5.089^**^	67.93 ± 4.66	71.02 ± 6.60
		3	67.64 ± 4.11^**^	52.67 ± 21.63^**^	50.50 ± 18.85^**^	69.82 ± 7.19^**^
		14	54.49 ± 14.63^**^	19.91 ± 9.06^**^	31.64 ± 20.05^**^	43.25 ± 21.21^**^

Means ± standard deviations for variables for each soil and litter type by day. Asterisks following means indicate significant differences between soil or litter types by day.

* P ≤ 0.05.

** P ≤ 0.01.

§ P ≤ 0.10.

a Values without standard deviations were for single observations.

Rinkes et al. (2013) reported significant effects of litter type, litter particle size, and soil type on CO_2_ efflux rates, but values of- and differences between litter and soil types were presented by litter particle size, making it difficult to compare their values to our estimates. Also, their rates were adjusted for soil-only control values and thus were slightly lower than we report. We found that although the total rate of CO_2_ efflux per g soil (μg C·g soil^−1^·d^−1^) was higher in loam than sand on all days, differences between litter types only occurred on day 3 when efflux was higher for maple than oak litter (Table 1). In contrast to rates per g soil, we found that rates of CO_2_ efflux per g TOC (mg C·g C^−1^·d^−1^) were not different between treatments on day 1, and rates were higher for sand than loam on both day 3 and day 14 (Table 1). On day 3 the CO_2_ efflux rate was also higher for maple than oak litter.

These differences in CO_2_ efflux rates per g soil and per g TOC can be explained by the large differences in SOC content between sand (ca. 0.4%) and loam (ca. 4.1%). Rinkes et al. (2013) found evidence of a priming effect for loam, suggesting that a portion of the C loss from loam was from the SOC pool rather than litter. However, SOC is likely more recalcitrant than litter, and because litter was a larger fraction of the TOC in sand than loam it likely supported a higher rate of respiration per g TOC. Apparently, differences per g soil between treatments were due to greater amounts of SOC and associated microbial biomass (see below) for loam than sand. Indeed, the higher CO_2_ efflux rate per g TOC on day 3 for sand may have resulted from the higher biomass: TOC ratio in sand on this same day (see below).

The biomass-specific respiration rates (g C·g C^−1^·d^−1^) were calculated for day 1 by dividing CO_2_ efflux rates on day 1 (μg C·g soil^−1^·d^−1^) by the microbial biomass on day 0 (μg C·g soil), which may have slightly overestimated rates because biomass was increasing during the first 24 h of the study (Table 1). Regardless, biomass-specific rates were higher for oak than maple on day 1. On other days there were no differences between treatments. This result also suggested a rapid convergence in metabolic characteristics of the microbial community driving C flux regardless of soil or litter type. In addition, CO_2_ efflux rates were higher for maple than oak litter on day 3, whether calculated per g soil or g TOC (Table 1), suggesting more rapid decay of the less recalcitrant maple litter even though it did not support a higher biomass: TOC value.

### Total carbon losses

Rinkes et al. (2013) subtracted the C lost from soil-only controls from their estimates of total C losses from treatments. They found consistent differences between soil and litter types, and occasionally differences between litter particle sizes. We did not subtract control values from treatments and found that cumulative CO_2_ efflux (mg C) was higher for loam than sand on all days (Table 1). On day 14, our mean values fell within the range of values reported by Rinkes et al. (2013) for the three particle sizes. We also found that C loss was higher for maple than oak on days 3 and 14 (Table 1). Not surprisingly, regressions showed that incubation time (day) explained most of the C loss in both loam (*N* = 14, *R*^2^ = 0.896; *P* = 0.01) and sand (*N* = 14, *R*^2^ = 0.860; *P* < 0.01). The overall loss rate in loam (0.00357 d^−1^) was 1.6 times greater than sand (0.00228 d^−1^). However, due to higher SOC content, loam lost <3% of its initial TOC by day 14 whereas sand lost about 5% (Table 1). There was also a slightly higher C loss (ca. 0.5%) from maple than oak litter in both soil types (both *P* ≤ 0.05).

Rinkes et al. (2013) found that the litter pool (ca. 0.44 g C) contributed most of the C to CO_2_ efflux because it was probably more labile than SOC, given a 5-month soil pre-incubation at optimal temperature and moisture conditions. Thus, the cumulative CO_2_ effluxes (calculated above) suggest that loam lost the equivalent of about 10% of its initial litter C by day 14 whereas sand lost only 7%. If the difference in C losses between sand and loam was due largely to the priming effect of litter addition on SOC turnover (Blagodatskaya and Kuzyakov, 2008), then the C loss from loam SOC (ca. 13 mg) approximated 0.6% of the initial SOC pool size.

Separate analyses by day revealed that the amount of estimated litter C remaining (mg C) on day 14 was greater for sand than loam, and greater for oak than maple (Table 1). This was consistent with larger cumulative C losses from maple than oak on days 3 and 14 for both soil types, and slightly higher rates of CO_2_ efflux for maple than oak on day 3, both per g soil and per g TOC (Table 1). This suggests a slightly higher rate of C acquisition from maple litter by microbial biomass, although no difference in biomass between litter types was observed at any time (see below). Rinkes et al. (2013) found that the priming effect was slightly higher for maple than oak litter, which might explain why biomass: TOC ratios did not increase with stimulation of recalcitrant SOC turnover (below), as the assimilation efficiency is likely lower for the more recalcitrant material.

### Microbial biomass

Microbial biomass per g soil (μg C·g soil^−1^) was initially greater for loam than sand. There were no differences between soil or litter types on day 3, but on day 14, biomass was again higher for loam than sand (Table 1). In contrast, microbial biomass per g TOC (mg C·g C^−1^) was greater for sand than loam on day 3, but there were no other differences between litter or soil types (Table 1). Apparently, differences that existed when biomass was estimated per g soil were due to the higher SOC and associated microorganisms present in loam than sand. When Rinkes et al. (2013) subtracted the amount of biomass in controls from treatments with added litter they found no differences between treatments at any time. However, they also estimated total biomass: TOC ratios and found higher values in sand than loam, and higher values on day 3 than day 14 for both soil types. These relationships between biomass and potential substrate (SOC and litter C) provide the basic parameters for models (Figure 1).

Most studies report that microorganisms usually account for less than 2-3% of the total organic matter in soils (e.g., Anderson and Domsch, 1989; Wardle, 1998), and would represent an even smaller fraction of the total soil mass. For this reason, the higher SOC content (and presumably associated biomass) for loam could explain the differences between soils in biomass per g soil on day 14 (Table 1). The biomass: TOC value on day 14 averaged 6.9 ± 5.6 mg C·g C^−1^ (ca. 0.7%) across all soil and litter types, suggesting a relatively consistent relationship regardless of soil or litter type. Nonetheless, we found that biomass: TOC ratios (mg C·g C^−1^) were higher for sand (2.5%) than loam (0.7%) on day 3, although there were no differences in biomass per g soil on day 3, nor were there differences in biomass: TOC on day 1. These results fell within observations by Rinkes et al. (2013) of 2.1–2.9% for sand and 0.7% for loam on day 3.

These results suggest an initial flush of microbial growth on fresh litter that was more apparent for sand than loam, because sand had a much lower SOC content. The much smaller amount of litter C (0.44 g C) supported a higher biomass: TOC ratio than the more recalcitrant SOC (0.20 g C for sand vs. 2.05 for loam) because C acquisition rate per g TOC (demonstrated by the difference in ratios between days 1 and 3), and thus per g biomass, was higher for sand. Wardle (1998) found that variation in soil biomass C across ecosystem types declined with increasing soil C, which is consistent with the differences between loam and sand we found in the present study. An unexpected result of this study was that there were no differences in microbial biomass estimates between litter types although oak is usually considered more recalcitrant than maple, and CO_2_ efflux was slightly higher for maple than oak (above).

### Enzyme activities

Extracellular enzyme activities can be expressed per unit soil, per unit organic matter, and per unit microbial biomass. Rinkes et al. (2013) subtracted soil-only control values from enzyme activities reported for treatments, to focus on litter activities. We examined litter + soil enzyme activities per unit soil mass and microbial biomass to focus on the whole system dynamics. For this reason, our activity values were much higher than those reported by Rinkes et al. (2013). We did not estimate activities per g organic matter because although the ratio of biomass: TOC (mg C·g C^−1^) was greater in sand than loam on day 3 (Table 1), no other significant differences in this ratio existed between treatments.

In general, Rinkes et al. (2013) found that enzyme activities per g soil increased over time, but with few treatment effects. In comparison, we found that all enzymes (not subtracting soil-only control values) showed higher activity per g soil (nmol ·g soil^−1^·h^−1^) in loam than sand on all days (Table 1). We also found that AP was higher for oak than maple litter on all days. In contrast, Rinkes et al. (2013) reported frequent interactions between litter particle size and soil type for AP, but consistently low activity for maple litter incubated in sand, and generally higher activity for loam than sand on day 14.

We found that β-glucosidase (BG) activity was higher for loam than sand on all days, whereas Rinkes et al. (2013) found that control-adjusted BG activities were higher for loam than sand only on day 14. We also found that BG was higher for maple than oak on days 0 and 3 (Table 1) whereas Rinkes et al. (2013) reported no difference between litter types.

Finally, we found that β-N-acetyl-glucosaminidase (NAG) activity was higher for loam than sand on all days, and for maple than oak on days 0 and 3 (Table 1). In contrast, Rinkes et al. (2013) found no differences in control-adjusted NAG activity between any treatments.

We calculated biomass-specific enzyme activities (μmol·g C^−1^·h^−1^) by dividing enzyme activities (nmol·g soil^−1^·h^−1^) by microbial biomass (μg C·g soil^−1^). There were no differences between soils or litters for any enzyme on day 0, when average activities were high but extremely variable (Table 1). On day 3, biomass-specific activities for AP were higher for loam than sand, and both BG and NAG were higher for maple than oak. On day 14, both BG and NAG were higher for sand than loam. These results contrast with higher activities of all enzymes per g soil for loam than sand on all days (Table 1). Although enzyme activities per g soil or g TOC are useful tools to evaluate treatment effects, biomass-specific enzyme activities are necessary to develop and test enzyme-based models (Figure 1), and as our results indicate, are not necessarily consistent with activities per unit soil. For example, the differences between enzyme activities per g soil on day 0 were apparently due to the higher initial microbial biomass for loam rather than differences in enzyme expression by microorganisms (Figure 1).

**Figure 2:**
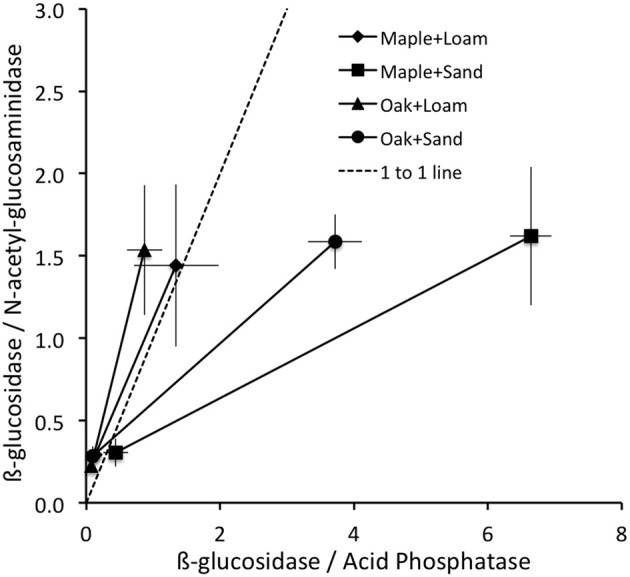
**Mean ± stdev ratios of enzyme activities were lowest on day 3 (closest to origin) and greatest on day 14 for all litter + soil combinations; 1:1 line shown**.

As an aide to interpreting patterns of enzyme activities, we followed Sinsabaugh et al. (2008) in calculating the ratios of BG/NAG and BG/AP activities for each pair of observations, including each combination of litter + soil treatment on each day (Figure 2). For each locus in this enzyme activity “space” we calculated an enzyme activity vector as the distance and angle from the origin. Vector length increases with increasing enzyme production toward C acquisition relative to nutrients (N and P), and the steepness of the vector angle increases with increasing enzyme production toward P acquisition. Thus we interpret increasing vector length as a relative increase in C limitation, and increasing vector angle as a relative increase in P vs. N limitation. The rationale for interpreting relative C, N, and P limitations to microorganisms from the relative activities of C, N, and P acquiring enzyme activities is based on stoichiometric and metabolic theories of ecological systems (Sterner and Elser, 2002; Gillooly et al., 2005; Allison et al., 2010, 2011). In brief, microbial requirements are relatively constrained by their elemental composition and metabolic demands, and needed resources are typically obtained from environmental sources through the actions of extracellular enzymes (Sinsabaugh and Follstad Shah, 2012). The ratios of BG/NAG are often plotted against BG/AP to determine the relative C, N, or P limitations to microorganisms, given the patterns of these key enzyme activities with respect to each other (Figure 2). Translating these ratios into vector lengths and directions (angles) provides clear metrics of relative C limitation (length), and relative P vs. N limitation (angle), but Rinkes et al. (2013) did not conduct these analyses.

Vector lengths were greater for maple than oak and greater for sand than loam on all days (Table 1). On day 0, vector angles were greater for sand than loam with no difference between litter types. In contrast, vector angles were greater for loam than sand and greater for oak than maple on both days 3 and 14 (Table 1). Vector lengths increased over time, and were significantly higher on day 14 than days 0 or 3 (Figure 2; all *P* = 0.01). Vector angles decreased over time, and were lower on day 3 than day 0 (*P* = 0.10), and lower on day 14 than days 0 and 3 (both *P* = 0.01). However, patterns differed by soil type. In sand, angles were significantly lower on day 3 than day 0, and lower on day 14 than day 3 (all *P* = 0.01). In loam, angles were only significantly lower on day 14 than day 3 (*P* = 0.01). In sand, lengths were higher on day 0 than day 3, and higher on day 14 than both days 0 and 3 (all *P* ≤ 0.05). In loam, lengths were only higher on day 14 than day 3 (*P* ≤ 0.05).

These patterns in enzyme activity vector length suggest that added litter provided a flush of soluble compounds driving biomass growth and concomitant immobilization of mineral nutrients, which could be obtained without enzyme activity. During this period of growth (day 0 to day 3), enzyme activity per unit biomass fell and relative C limitation declined (shorter vector lengths), although this pattern was stronger for sand than loam. By day 14, readily available soluble compounds from litter and mineral nutrients from soil may have been depleted and biomass-specific enzyme activities increased (along with vector lengths; Figure 2). Angles declined over time for both soil and litter types, but changed more for oak than maple litter (Figure 2, Table 1), suggesting that microorganisms became relatively more N limited for oak over time.

### Integrating enzyme activity, CO_2_ efflux, and microbial growth

We analyzed the incremental growth of microbial biomass (μg C·g soil^−1^) over time, which we calculated as the difference between sequential observations for each combination of litter, soil, and litter size treatments (Table 1). Cumulative enzyme activity (μmol·g soil^−1^) was calculated by multiplying the average activity of an enzyme between dates by the time span. Biomass growth estimates were compared to the lengths and angles of enzyme activity vectors at the time of observations (above), the cumulative activities of enzymes between observations, and the cumulative CO_2_ efflux (mg C) during the same periods. Stepwise regression showed that the length of the enzyme activity vector and cumulative acid phosphatase (∑AP) activity together explained most of the variation in microbial growth, and that growth was negatively related to both factors (Table 2). Thus, our results suggest that greater microbial growth was associated with lower microbial investment in C and P acquisition via enzyme production.

**Table 2:** **Regression coefficients relating the length of the enzyme activity vector and cumulative acid phosphatase activity (∑AP; μmol·g soil) to change in biomass (*N* = 24, *R*^2^= 0.722)**.

**Variable**	**Coefficient**	**Standard error**	***P*≤**
Intercept	298.11	42.75	0.01
Length	−77.84	11.62	0.01
∑AP	−1.35	0.32	0.01

We also compared cumulative respiration (∑CO_2_) to cumulative enzyme activity (∑NAG, ∑BG, and ∑AP). The underlying assumption for this comparison was that respiration is an index of microbial metabolism fueled by the actions of extracellular enzymes, and thus should be related to enzyme activity. The only direct measure of microbial activity in this study was CO_2_ efflux, but other studies have shown cumulative enzyme activity to be positively correlated with various measures of decomposition (Sinsabaugh, 1994; Jackson et al., 1995; Amin et al., 2013). We found significant relationships between ∑CO_2_ and both ∑NAG and ∑BG, with no significant effects of litter or soil type (Table 3). These consistent relationships between C and N acquiring enzyme activities across litter and soil types suggest that these relationships were very conservative within the constraints of this study.

**Table 3:** **Regressions of cumulative CO_2_ efflux (mg C) against the cumulative activities of acid phosphatase (AP), β-N-acetyl-glucosaminidase (NAG) and β-glucosidase (BG) (μmol)**.

**Parameter**	**BG**	**NAG**	**AP**
*N*	28	28	28
Intercept	18.34	13.43	18.16
Slope	0.368	0.467	0.234
*R*^2^	0.642	0.833	0.657
*P*≤	0.01	0.01	0.01

**Figure 3:**
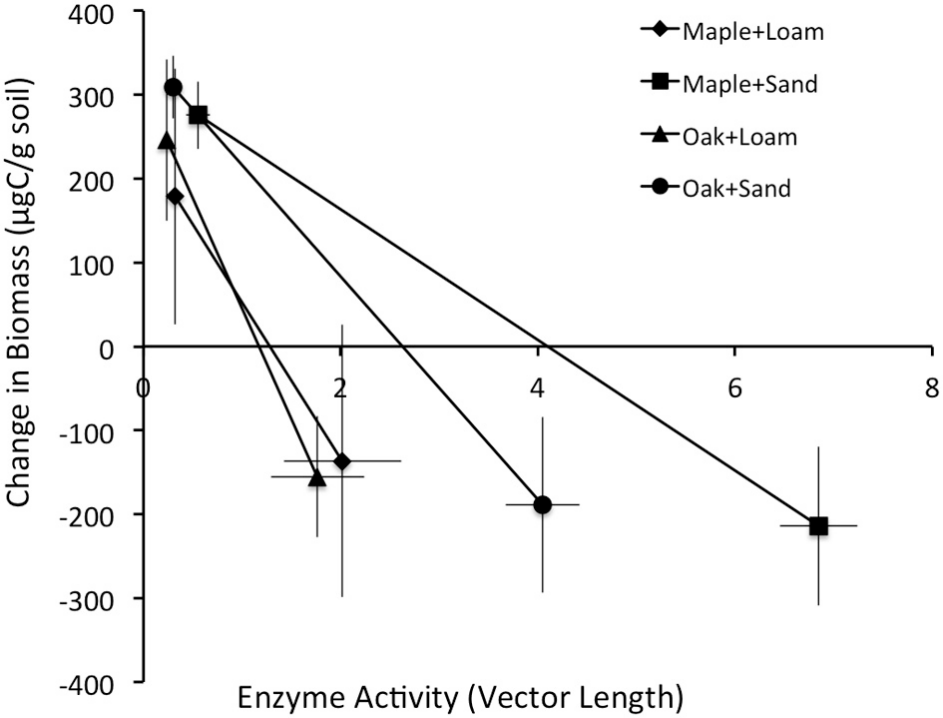
**Mean ± stdev change in biomass (μg/g soil) vs. the length of the enzyme activity vector (relative C vs. nutrient limitation)**.

In contrast, the relationship between ∑CO_2_ and ∑AP was highly variable over time and between litter and soil types. Our analyses showed significant effects of both soil and litter types as well as interactions between all factors, suggesting complex, inconsistent relationships between microbial respiration and AP activity. Overall, these systems appeared to be more strongly P than N limited between days 0 and 3 (vector angles >45°C; Table 4), especially for oak and loam (Figure 2, Table 1), but became more N limited by day 14 (vector angles <45°C; Table 4), especially for sand (Figure 2, Table 1). In comparison, the change in microbial biomass between days 3 and 14 was negatively related to both the vector length and cumulative acid phosphatase (∑AP) activity (Figure 3, Table 4). In fact, microbial growth was negatively related to the cumulative activity of each enzyme (not shown), which were highly correlated with one another. In essence, the negative relationship between growth and vector length also suggested an increasing C limitation over this time period (Figure 2, Table 4). The negative relationship between growth and cumulative enzyme activity suggests that less growth may occur when resources become more limiting and require an increase in relative enzyme production.

**Table 4:** **Mean ± stdev values for enzyme activity (BG, β glucosidase; NAG, β-N-acetyl-glucosaminidase; AP, acid phosphatase; mmol·g C^−1^·h^−1^), enzyme activity vector length (mol·mol^−1^) and angle (degrees), and microbial biomass (mg C·g C·h^−1^)**.

**Parameter**	**Day 0**	**Day 3**	**Day 14**
BG	5.98 ± 8.78ab	0.19 ± 0.13a	5.44 ± 4.13b
NAG	4.15 ± 4.92b	0.72 ± 0.43a	3.41 ± 2.26b
AP	16.05 ± 28.83b	1.53 ± 1.22a	2.51 ± 1.86a
Vector length	1.35 ± 0.86a	0.35 ± 0.14a	3.67 ± 2.16b
Vector angle	70.29 ± 5.79a	60.44 ± 17.26a	36.12 ± 20.63b
Biomass	2.38 ± 1.51a	16.41 ± 9.79b	6.95 ± 5.35a

Different letters following means within rows indicate significant differences between days.

### Mineral nutrient concentrations

Rinkes et al. (2013) reported that prior to litter addition all soil properties differed between soil types except for pH and initial PO^−3^_4_ concentrations. However, we found that immediately after litter addition (within 30 min), PO^−3^_4_, NO^−^_3_ and NH^+^_4_ concentrations (μg·g soil^−1^) varied between both soil types and litter types, probably due to differences in nutrient contents of litter. PO^−3^_4_ was significantly higher for sand than loam for all days (Table 1), as reported by Rinkes et al. (2013). However, we also found that PO^−3^_4_ was higher for maple than oak litter on all days.

Our analyses showed that NO^−^_3_ and NH^+^_4_ were higher for loam than sand on all days (Table 1), as reported by Rinkes et al. (2013). However, Rinkes et al. (2013) reported low overall concentrations of NH^+^_4_ for sand (<5 μg·g soil^−1^) throughout the experiment, but high initial concentrations for loam (15–30 μg·g soil^−1^) falling to <5 μg·g soil^−1^ by day 14, resulting in no differences between soils on day 14. We also found that NH^+^_4_concentrations were higher for oak than maple litter on all days (Table 1), whereas Rinkes et al. (2013) reported higher control-adjusted concentrations for oak than maple only on day 0.

Another interesting result of this study was that neither the concentrations nor the dynamics of soil PO^−3^_4_, NO^−^_3_, and NH^+^_4_ pools provided much explanation for the dynamics of system C, microbial biomass, or enzyme dynamics. AP had a consistent, positive relationship to NH^+^_4_ (not shown), but it was the only measure of enzyme activity that showed any consistent relationship to any soil nutrient. Overall, the vector angle of enzyme activity was positively related to NO^−^_3_, and NH^+^_4_, but this relationship varied by day.

## Insights to key relationships

The primary goal of this paper is not to elucidate the effects of soil and litter types on decomposition processes, *per se*, but to illustrate how the interpretation of experimental data from a modeling perspective could differ from other approaches. We also tried to highlight the importance of collecting simultaneous, comparative measures of key model features, such as microbial biomass, enzyme activity, and respiratory output, by examining such data from the study by Rinkes et al. (2013). For example, many differences in microbial biomass, CO_2_ efflux rates, and enzyme activities that existed between litter and soil types when observations were expressed per g soil were not apparent or were different when they were expressed per g TOC or per g microbial biomass (Table 1). Moreover, the relationships between enzymes, resources, and microorganisms were fairly tightly constrained, consistent with the theory of ecoenzymatic stoichiometry (Sinsabaugh and Follstad Shah, 2012).

Regardless of the reason why values of biomass: TOC were constrained (Wardle, 1998) any limit to biomass necessarily limits extracellular enzyme activities because they are produced by microorganisms and are likely to be related to other microbial activities (e.g., CO_2_ efflux). Although many factors control the persistence and activity of enzymes in the environment (Nannipieri and Gianfreda, 1998; Nannipieri et al., 2012; Burns et al., 2013), Sinsabaugh and Moorhead (1997) argued that a more rapid turnover of microbes than their enzymes would lead to an unstable system. Similarly, the model by Allison (2005) suggests that decoupling enzyme activity from the microorganisms that produced them would permit other microorganisms (i.e., “cheaters” that don't produce enzymes) to potentially destabilize the system. Within this context, the observed biomass-specific respiration and enzyme activities represented the boundaries of these constraints for this study system. For example, microbial biomass: TOC was higher for sand (maximum 2.5%) than loam (maximum 0.7%) because the TOC of sand (0.64 g) was dominated by litter (0.44 g) whereas the TOC of loam (2.49 g) was dominated by more recalcitrant SOC (2.05 g). Thus the higher relative C availability in sand supported a higher relative biomass per unit substrate C, but only on day 3; by day 14 the ratio no longer differed between soils. This pulse of biomass growth for sand also provides an explanation for patterns of enzyme activity.

The patterns of biomass-specific enzyme activities indicated few differences in C, N, and P acquisition between soils or litter types. BG activity was higher for sand than loam on day 14, consistent with a greater enzyme activity vector length (Table 1), suggesting a higher relative C availability for loam. BG activity also was higher for maple than oak on day 3, again consistent with a greater vector length. AP activity was greater for loam than sand on day 3, suggesting a greater P demand for loam. Vector angle was also higher for loam than sand on day 3, also consistent with greater P vs. N demand. Finally, NAG was higher for oak than maple on day 3, and higher for sand than loam on day 14, suggesting greater N demands. Vector angles were lower for both of the latter cases, consistent with higher N vs. P demands. Thus patterns in biomass-specific enzyme activities were consistent with characteristics of enzyme vectors although vectors provided more detailed insights to microbial demands for C, N and P. For example, vector lengths indicated greater C than N or P limitation for sand on all days, suggesting that microorganisms were consistently more C limited in sand than loam, despite few differences in BG activities. In sand, the significant decrease in biomass: TOC between days 3 and 14 (not shown) suggested that increasing C limitation may have been responsible for the decline in biomass. At the same time, vector length increased 10-fold (Table 1). Vector angles indicated initially greater P vs. N limitation for sand than loam (day 0), but changed to a greater N vs. P limitation in sand for both days 3 and 14 (Figure 2, Table 1). Angles also indicated consistently greater P vs. N limitation in oak vs. maple litter. In contrast, biomass-specific AP and NAG activities showed few differences between litter or soil types. This pattern in vector angles was consistent with the higher PO^−3^_4_ for sand and maple litter on days 3 and 14, concomitant with higher NO^−^_3_ and NH^+^_4_ for loam than sand on all days (Table 1).

Understanding enzyme-biomass relationships needed for mechanistic models (Figure 1) requires understanding biomass-resource relationships that control the allocation of enzymes toward C, N, and P-acquisition (Sinsabaugh and Follstad Shah, 2012). Thus it is not simply the activities of enzymes in the environment or even the biomass-specific activities, but the balance between activities like those revealed herein by enzyme vectors. Recently, Moorhead et al. (2012) were able to simulate differential allocation of C and N acquiring enzyme activities during decomposition in response to relative C and N availability by assuming that enzyme production was finite and that microorganisms optimized resource acquisition to maximize growth. The observations of Rinkes et al. (2013) verify these general assumptions, but require analysis of relational variables, such as C, N, and P acquiring enzyme activities to describe this balance.

This study also refutes a common assumption of enzyme-based models that enzyme production is roughly constitutive (Schimel and Weintraub, 2003; Moorhead et al., 2012), because a decline in enzyme activity occurred after the addition of fresh litter to microcosms despite an increase in biomass. Thus, biomass-specific activities of all enzymes were generally lowest on day 3 although biomass was greatest on day 3. If enzyme production were strictly constitutive, activity would increase with biomass. Instead, there appeared to be a shift in patterns of substrate use, with fresh litter driving a flush of CO_2_ efflux and microbial growth. Enzyme activities then increased from day 3 to 14 despite a decline in biomass (Figure 2, Table 1), also suggesting a change in substrate use and changing emphasis on enzyme production. These results suggest that enzyme production was inducible, as modeled by Allison (2005), and responsive to differences in substrate characteristics (Berg, 2000; Berg and McClaugherty, 2008). In brief, microorganisms initially use simpler, easier to obtain resources from soluble litter fractions, shifting to increasingly more recalcitrant compounds as decomposition progresses (Van Hees et al., 2005; Glanville et al., 2012). This pattern also is consistent with the idea that different groups of microorganisms may be more active at different stages of litter decay (Allison and Martiny, 2008; Rinkes et al., 2011), including an initial burst of activity by some that may have little to no enzyme production (Allison, 2005).

Relationships between CO_2_ efflux, biomass, and enzyme activities also addressed key model behaviors (Figure 1). For example, biomass-specific CO_2_ efflux rates ranged from 32–85% of the standing biomass C per day (Table 1), with no differences between soil or litter types or any difference between day 3 and day 14. Thus the respiratory coefficient was consistent despite differences in B:C ratios between soils on day 3 and despite the very different total amounts of biomass in the two soil types (Table 1). A respiratory coefficient of this magnitude is much higher than basal metabolic rates of usually <1% used in models (Parton et al., 1987; Skjemstad et al., 2004; Moorhead and Sinsabaugh, 2006), suggesting high growth-associated respiration and turnover rates, and possibly overflow metabolism to maintain observed biomass: TOC ratios (e.g., Parnas, 1975; Schimel and Weintraub, 2003). However, Rinkes et al. (2013) did not apply an extraction efficiency coefficient (K_ec_) to the amounts of microbial biomass C they extracted with chloroform fumigation. Thus their biomass estimates are probably low, which would inflate the respiratory coefficient.

We also found that ratios of ∑CO_2_-efflux: ∑enzyme activity declined through time, suggesting a decline in enzyme efficiency, although a progressive change in substrate composition and selective use by microorganisms could also contribute to this pattern (see above). Moreover, this ratio is an ambiguous measure of enzyme efficiency (Sinsabaugh, 1994; Jackson et al., 1995), particularly when gross measures of decomposition are used in calculations (e.g., CO_2_-efflux), because enzymes usually target a specific type of substrate. It would be a more accurate measure of enzyme efficiency to relate changes in specific substrates, like cellulose, to the activities of enzymes that degrade them, like β-glucosidase (e.g, Amin et al., 2013). As an aside, significant positive intercepts from regressions of cumulative decay (e.g., ∑CO_2_ efflux) vs. cumulative enzyme activity are also consistent with the idea that initial decay is relatively high for low levels of enzyme activity (Table 3), again arguing for changing substrate use patterns. These empirical data suggest that enzyme-based models will need to include multiple substrates with inducible enzyme activities to accurately portray SEM dynamics (Figure 1).

In summary, much of the information used to develop enzyme-based models to date has been obtained from disparate studies, which separately focused on various aspects of decomposition, microbial metabolism, enzyme activities, etc. (e.g, Schimel and Weintraub, 2003). Although insights can be gained from cross-system analyses, uncertainty about key relationships is a drawback. For example, relatively few studies have examined biomass dynamics during litter decay with much resolution (but see Fioretto et al., 2001), which is critical to linking enzyme activities to decomposition processes. More comprehensive studies like the one performed by Rinkes et al. (2013) reduce uncertainty by simultaneously measuring key variables that in turn permits direct comparisons, as shown herein. Moreover, Rinkes et al. (2013) also measured respiration for control soils (no litter) and control litter (no soil) in parallel incubations (not shown), so that the system dynamics of litter, soil and litter + soil could be isolated, although a significant priming effect found for loam demonstrated a synergism between litter and soil systems (Kuzyakov, 2010).

## Conclusions

Despite the information gained from this detailed study, other relationships needed to inform enzyme-based models remain unknown (Burns et al., 2013). Among the more important are turnover rates for both enzymes and biomass, which presumably enter the SOC pool and thus become available for decomposition. Also unknown is the relationship between enzyme activity, concentration, and mass. Although Wang et al. (2012) recently suggested ways of estimating kinetic coefficients for enzymes in the field from laboratory studies, the relationship between activity and concentration is uncertain and highly variable, due to the influences of many environmental factors (Nannipieri and Gianfreda, 1998; Nannipieri et al., 2012). Models that presume to calculate enzyme pool sizes (e.g., Sinsabaugh and Moorhead, 1997; Schimel and Weintraub, 2003; Allison, 2005; Moorhead et al., 2012, etc.), in fact balance allocation, production, turnover and resource acquisition without direct observations. Such observations are needed to more directly and precisely determine the cost-benefit relationships of microbial investments in extracellular enzymes.

Another important mechanism underlying decomposition not mentioned herein is the taxonomic composition of the microbial community. Different microorganisms have different environmental responses, enzyme capabilities, and metabolic and stoichiometric characteristics (Berg and McClaugherty, 2008). Thus all three of the substrate, enzyme, and biomass pools in Figure 1 must expand to capture the more complex realities of the SEM relationships driving decomposition, which greatly increases the demand for experimental data (see Moorhead and Sinsabaugh, 2006). As previously discussed, few studies have measured key features of the simple enzyme-based model represented in Figure 1. Even fewer have examined the composition of the microbial community, and possibly no studies have obtained information on changes in key system characteristics (e.g., Figure 1) corresponding to community changes. This work is only beginning.

### Conflict of interest statement

The authors declare that this research was conducted without any commercial or financial relationships that could be construed as a potential conflict of interest.
